# Neuromyelitis Optica Spectrum Disorder Mimicking Pontine Stroke: A Case Report and Systematic Literature Review

**DOI:** 10.7759/cureus.41099

**Published:** 2023-06-28

**Authors:** Brendan Huang, Rohan Arora, Spencer McFarlane, Joseph A Diamond, Souhel Najjar

**Affiliations:** 1 Neurology, Northwell Health, Manhasset, USA

**Keywords:** anti-aqp4 autoantibody, myelin-oligodendrocyte glycoprotein (mog), neuromyelitis optica spectrum disorder (nmosd), mimic disease, systematic literature review, neuromyelitis optica spectrum disorder, stroke

## Abstract

Neuromyelitis optica spectrum disorder (NMOSD) is a rare autoimmune disorder that was first described in the late 1800s as a variant of multiple sclerosis (MS). However, it has recently been categorized, as a disease, especially with the discovery of aquaporin-4 (AQP4-Ab) and myelin oligodendrocyte glycoprotein antibodies (MOG-Ab). Unfortunately, patient presentation is not always clear, and NMOSD may initially be diagnosed as an alternative neurological disease. We present a 58-year-old woman who was hospitalized several times for what was initially perceived as a pontine stroke. However, given worsening symptoms, serologic testing confirmed AQP4-Ab positivity and, subsequently, the NMOSD diagnosis. In addition to the case report, a systematic literature review was performed to identify NMOSD cases initially misdiagnosed as stroke. Publications were selected and curated in accordance with Preferred Reporting Items for Systematic Reviews and Meta-Analyses (PRISMA) guidelines. Six NMOSD patients were initially thought to have had acute strokes. However, steady progression and/or the recurrence of symptoms suggested that further investigations with neuroimaging studies and serological immune assays were necessary to exclude alternative etiologies. Notably, the age at onset in all cases was significantly more advanced than patients with typical NMOSD presentations (median age 32-41). In conclusion, the NMOSD diagnosis should be considered in cases with atypical stroke-like presentations, particularly those of later onset (defined as equal to or greater than 50 years of age). This is important as early recognition and treatment with immune therapies can improve functional outcomes.

## Introduction

Neuromyelitis optica (NMO) is a rare autoimmune disorder with significant morbidity and mortality, particularly among those with delayed diagnosis and treatment. It was first reported in the late 1800s to describe a severe demyelinating syndrome characterized by optic neuritis and myelitis. Until 2004, NMO was thought to be a severe variant of multiple sclerosis (MS) [1]. In 2004, aquaporin-4 antibodies (AQP4-Ab) were identified in the majority of patients with the clinical syndrome of NMO, ultimately confirming that NMO is a unique entity distinct from MS [2]. NMO is a much rarer disease, with an estimated prevalence of approximately 2-4/100,000 in most populations [3]. Patients of Hispanic and African descent are more frequently affected. Classically, NMO was defined as a condition that spared the brain itself, but it is now generally accepted that NMO lesions can affect the brainstem and cerebrum [4-5]. Currently, neuromyelitis optica spectrum disorder (NMOSD) is the more commonly used term to reflect the broad spectrum of clinical presentations subclassified according to their serological status. The three subclassifications are myelin oligodendrocyte glycoprotein antibody (MOG-Ab) seropositive, AQP4-Ab seropositive, and AQP4-Ab seronegative. Here, we describe the case of a Hispanic woman who presented with right hemiparesis and an acute left pontine lesion mimicking an ischemic stroke. However, the atypical progressive neurological course, together with the unexplained gastrointestinal manifestations, provided clues as to alternative etiologies. Further diagnostic workup confirmed the diagnosis of NMOSD.

## Case presentation

**Figure 1:**
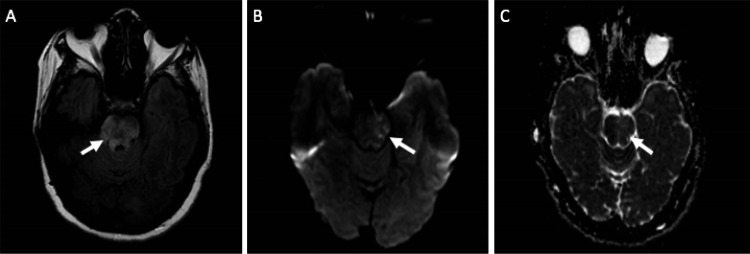
MRI scans from the initial presentation. Arrows in each panel indicate the pontine region of interest. (A) Axial T2 fluid attenuated inversion recovery imaging (B) Axial diffusion weight imaging (C) Axial apparent diffusion coefficient imaging

A 58-year-old Hispanic woman presented to one of our in-network hospitals with bilateral blurry vision, decreased sensation in the left V2 distribution, and right hemiparesis. Approximately three weeks earlier, she was treated at an outside hospital for vertigo, right-sided numbness, and weakness and was found to have a pontine lesion initially interpreted to be an acute infarct on neuroimaging. Her National Institute of Health Stroke Scale (NIHSS) score was 1. Thrombolytics were not given as the patient was outside the window. On the neurological exam, she was alert and oriented to person, place, and time. She did not exhibit aphasia, dysarthria, or dysphagia. Motor strength was 5/5 on the left upper and lower extremities but 4/5 on the right upper and lower extremities. Light touch sensation was slightly reduced in the right thigh region but otherwise intact in all other distributions. Muscle stretch reflexes were 3+ and symmetric throughout. No appendicular, truncal, or gait ataxia was noted. Brain MRI imaging demonstrated a left pontine lesion, initially thought to represent acute infarction (Figures 1A-1C).

The patient was subsequently discharged on dual antiplatelet therapy, aspirin and clopidogrel, and atorvastatin for secondary stroke prevention.

**Figure 2:**
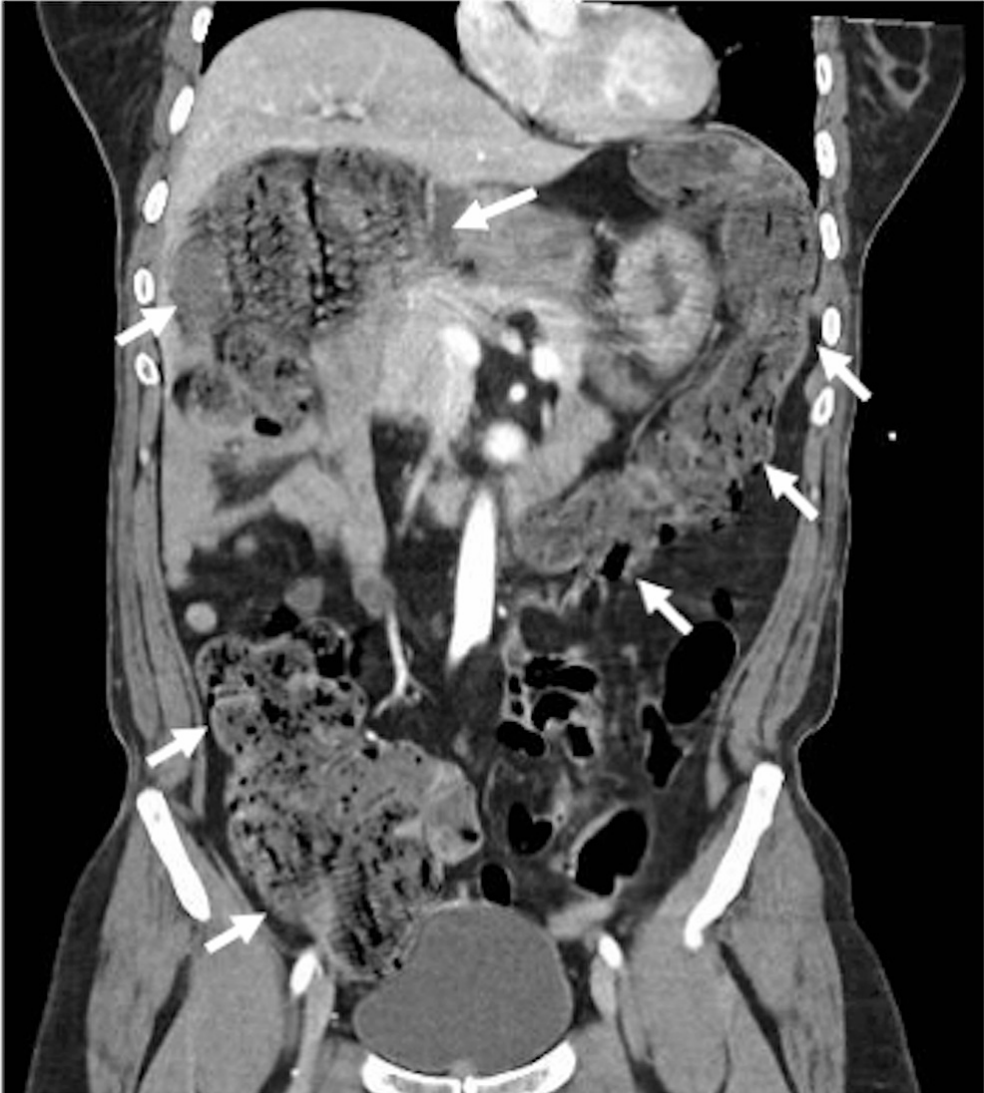
Abdominal CT scan. Arrows demonstrating the extent of a distended colon. An axial scan demonstrated packed feces during the course of the hospital stay, which was suggestive of constipation

Several days later, the patient was evaluated at a different in-network emergency room for mild shortness of breath and significant abdominal discomfort with decreased bowel movement. An abdominal computed tomography (CT) scan revealed packed stools without bowel obstruction (Figure 2).

**Figure 3:**
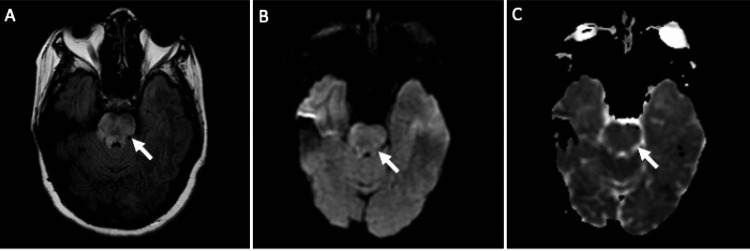
MRI scans from the second presentation. Arrows in each panel indicate the pontine region of interest. A) Axial T2 fluid attenuated inversion recovery imaging (B) Axial diffusion weight imaging (C) Axial apparent diffusion coefficient imaging

She was started on laxatives and discharged. A few days later, she was admitted to our tertiary care hospital for new-onset generalized weakness with worsening right hemiparesis, dysarthria, right sixth nerve palsy-related horizontal diplopia, right facial droop, and tinnitus. On neurological examination, she had a dysconjugate primary gaze due to right sixth nerve palsy, dysarthria, generalized weakness more pronounced on the right side (4-/5), and slightly reduced sensation of the right leg. She had a slight right-hemiparetic gait. Brain MRI demonstrated restricted diffusion lesion on diffusion-weighted imaging (DWI) and fluid-attenuated inversion recovery (FLAIR) in the periventricular white matter and brainstem region, suggesting a possible rhombencephalitis of inflammatory or autoimmune origin (Figures 3A-3C).

The serological immune assay was notable for positive AQP4-Ab titers of 1:1000. Cerebrospinal fluid (CSF) analysis showed lymphocytic pleocytosis (20 white blood cells with 97% lymphocytes) with normal protein (19 mg/dL; normal range 15-45) and glucose (58 mg/dL; normal range 40-70) levels. There were two unique CSF oligoclonal bands: high CSF AQP4-Ab titers (1:32; normal <1:2) and absent CSF MOG-Ab. Myelin basic protein (MBP) was elevated (27.1 ng/mL; normal range 0.0-3.7). Infectious assays were negative, including those for acid-fast bacilli, fungus, viral pathogens (including herpes simplex viruses 1 and 2, Epstein-Barr virus, and West Nile Virus IgG and IgM), and cryptococcal antigen.

**Figure 4:**
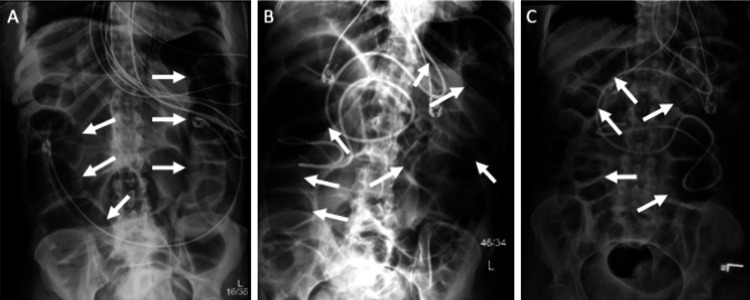
Abdominal X-ray imaging of the patient throughout the hospital course. Arrows demonstrating the level of colonic distension and subsequent compression (A) Acquired several days after admission into the medical intensive care unit, demonstrating marked gaseous extension of the colon; (B) Worsening gaseous distention of the small and large bowel, correlating with hyperactive abdominal and pseudo-obstruction. (C) Decreased abdominal distention after nasogastric and rectal tube placement

Accordingly, the patient was diagnosed with NMOSD and started on intravenous immunoglobulin therapy (IVIG) at 2g/kg/day. Despite IVIG infusion, the patient’s condition deteriorated rapidly, with worsening abdominal pain and bulbar symptoms. She developed respiratory distress, was intubated for airway protection, and was transferred to the medical intensive care unit (MICU). Her neurological examination was also notable for generalized weakness, as follows: 3/5 strength of both upper extremities and 2/5 strength of both lower extremities. She was found to have a severe ileus that required a temporary jejunostomy feeding tube placement (Figures 4A-4C).

The patient was subsequently started on methylprednisolone pulse dosing of 1 gram intravenous daily for five days, followed by a tapering course of oral corticosteroids. Five days after IVIG, the patient was started on five days of plasma exchange, followed by a rituximab course of two doses of 1000 mg IV infusion separated by two weeks. These treatments resulted in substantial improvements in her neurological and medical conditions. She was extubated, and the ileus resolved. Upon discharge to the acute rehabilitation facility, she was widely awake, alert, and able to follow simple commands. The speech was hypophonic but fluent. Right-six nerve palsy and facial weakness have resolved nearly fully. Her muscle strength was as follows: 4-/5 of proximal left upper extremities, 4+/5 of distal left upper extremities, 3/5 of proximal right upper extremities, 4+ of distal right upper extremities, 3/5 in proximal and distal left lower extremities, 4-/5 of proximal right lower extremities, and 4+/5 of distal right lower extremities. About one month later, she was noted to have normal mentation with intact language and memory functions. The speech was fluent, with no dysarthria. Cranial nerves were normal, including extraocular movements and facial strength, and tone. She had a full strength of 5/5 in arms and legs with normal tone and bulk. Notably, ileus and abdominal pain have completely resolved.

## Discussion

Methods

**Figure 5:**
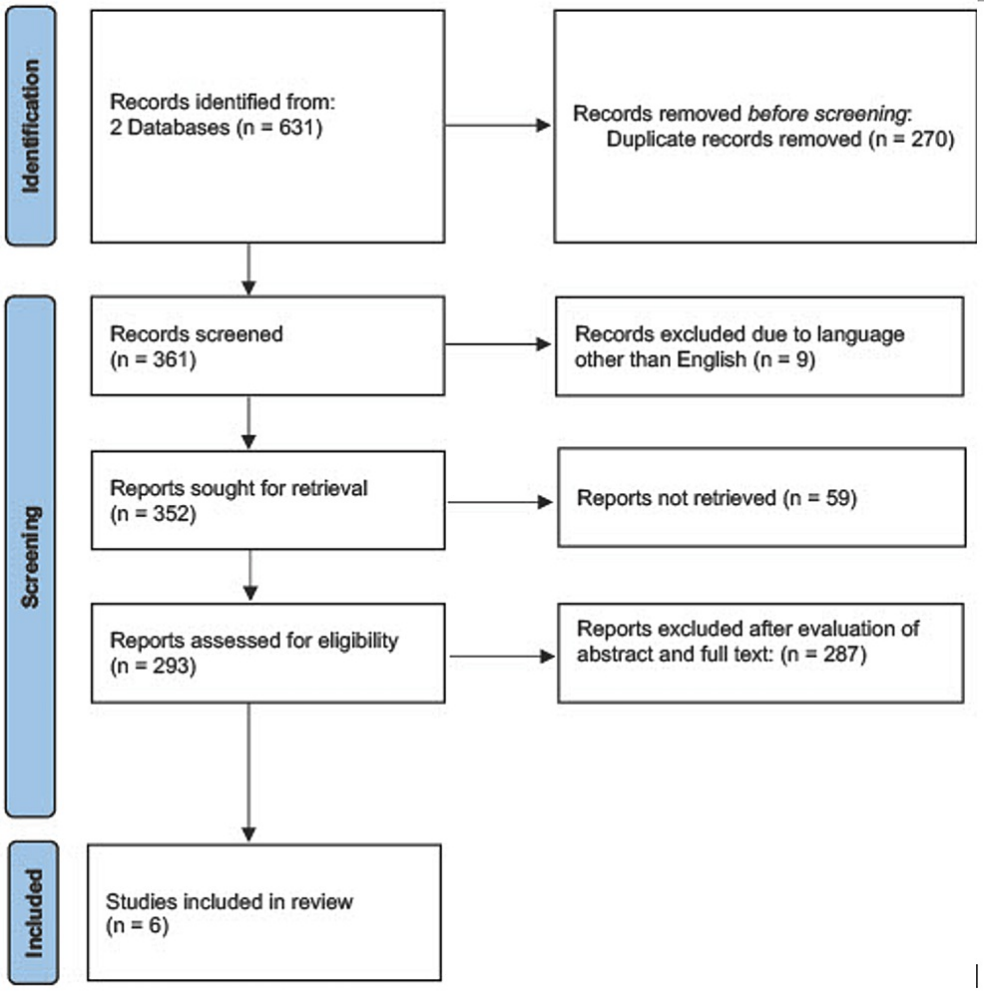
Diagrammatic representation of literature searches utilizing PubMed and the Web of Science Database

A comprehensive search was completed utilizing the Preferred Reporting Items for Systematic Reviews and Meta-Analyses (PRISMA) guidelines. Titles and abstracts between 2007 and 2022 were identified via the PubMed database, while the article dates were set between 2002 and 2022 via the Web of Science (WoS) database. Only one article was found outside of the time parameters, published in 1993, and was deemed not applicable to the current systematic review [6]. The following Boolean search terms were used: “Neuromyelitis optica spectrum disorder” AND “stroke” in addition to “Neuromyelitis optica” AND “stroke.” References from selected articles were assessed for relevant works. Exclusion criteria included studies written in a language other than English, review articles, studies where full text was unable to be acquired, animal and in vivo models, and those irrelevant to NMOSD and stroke. The results of the electronic search were included in the EndNote web-based bibliographic management software (Figure 5). In total, six case reports were selected after a thorough analysis of abstracts and full-text reviews and utilized by the authors of this publication.

Six case reports detailing six patients were accumulated into a table, listing their demographics, presenting symptoms, initial diagnosis, imaging modality, continuing presentation, final diagnosis, and eventual treatment [7-12]. The seventh case, detailing the current case presentation, was included.

Results

Using PubMed with the search terms “Neuromyelitis optica” AND “stroke” as well as “Neuromyelitis optic spectrum disorder” AND “stroke,” 95 and 95 articles were found, respectively. Using the Web of Science with the same two search terms, 329 and 112 articles were found, respectively. When all articles were combined into the EndNote bibliographic database and duplicate records were removed, 361 articles remained. Nine articles were eliminated due to a non-English language being the predominant language for the article. Fifty-nine references were not included due to the full text not being able to be acquired. Afterward, 287 articles were eliminated due to not meeting inclusion criteria, resulting in six full-text articles. Titles and abstracts for the 59 references with no full-length articles were perused, and none met the inclusion criteria (Figure 5).

Data analysis

Seven patients were evaluated, six from previous case studies and our current case presentation. The median age of patients was 58 (Range 52-84). There was a 6:1 female: male predominance, with the sole male being presented by Li et al. [10]. Four patients presented with a form of hemiparesis stemming from a suspected anterior circulation stroke [8,10-12], whereas a minority presented with non-focal symptoms such as vomiting, gait instability, and blurry vision, implying a possible posterior circulation stroke. Only one patient presented with hemorrhage, which was likely an ischemic stroke with hemorrhagic conversion [8]. MRI imaging of the brain and spine was the predominant imaging modality that was used to diagnose stroke and, later, NMOSD. Serum AQP4-Ab was the predominant method used to diagnose NMOSD, whereas oligoclonal bands were only present in one patient [7]. Methylprednisolone was the initial medication of choice for the treatment of NMOSD, with 1g/day being the standard dose. Only one patient was treated with less than 1g/day of methylprednisolone [10]. Two types of steroid tapers were used: those that converted to an oral steroid and those that continued to provide intravenous methylprednisolone but at a lower frequency. Several types of immunosuppressants were used: azathioprine, mycophenolate mofetil, and rituximab [7,10]. Pneumonia (PNA) was the most predominant complication, with one patient eventually succumbing at follow-up [9,10]. 

Discussion

NMO is a rare autoimmune disorder with significant morbidity and mortality, particularly among those with delayed diagnosis and treatment. This case underscores the importance of considering alternative etiologies such as NMO in the differential diagnosis of atypical stroke syndrome presentations. The diagnosis of neuromyelitis optica and its variants is defined by the International Panel for NMO Diagnosis (IPND). [13]. The recent guidelines stratify NMOSD based on serological status AQP4-Ab, whether positive or negative. Studies by Hamid et al. demonstrated that about one in four patients with an NMOSD diagnosis is AQP4-Ab seronegative [14]. The addition of CSF studies to serological studies for NMO diagnosis is debatable. Klawiter et al. described three NMO patients in whom CSF studies of AQP4-Ab were positive while serum AQP4-Ab was negative [15]. However, Jarius et al. found that CSF studies did not significantly boost the sensitivity and specificity of NMO diagnosis [16]. Furthermore, seropositivity with dilutions greater than 1:250 was associated with acute cases of NMO [16]. In our case, the detection of AQP4-Ab antibodies in the serum and CSF at 1:1000 and 1:32 dilutions, respectively, is consistent with findings reported by Jarius et al. and confirms the diagnosis of NMO [13]. Clinically, the recurrence and progression of the neurological symptoms and deficiencies, together with the comorbid unexplained gastrointestinal manifestations (such as significant abdominal discomfort, severe constipation, and ileus requiring a temporary jejunostomy feeding tube placement), provide additional clues to the alternative etiologies of the brainstem seemingly stroke presentation, such as neuroinflammatory processes, and the final confirmation of NMOSD. Indeed, ileus has been found in several case studies of patients who were eventually diagnosed with NMOSD [17-19].

Our systematic review identified six additional case reports, summarized in Table 1. 

**Table 1:** A literature review of cases, including the present study, where NMOSD was initially diagnosed as stroke M: male. F: female, V: Vomiting, MRI: Magnetic resonance imaging, L: Left, T: Thoracic, OB: Oligoclonal band, AQP4-Ab: Antibodies to aquaporin-4, CSF: Cerebrospinal fluid, NMOSD: Neuromyelitis optica spectrum disorder, mPRED: Methylprednisolone, g: Gram, d: Day, AZA: Azathioprine, R: Right, CT: Computed tomography, MRA: Magnetic resonance angiography, ICA: Internal carotid artery, ACA: Anterior cerebral artery, MCA: Middle cerebral artery, UE: Upper extremity, ANA: Antinuclear antibody, SS-A: Anti-Sjögren’s syndrome type A, SS-B: Anti-Sjögren’s syndrome type B, NMO: Neuromyelitis optica, b.l.: Bilateral, LE: Lower extremity, N: Nausea, HA: Headache, VA: Vertebral artery, N/A: Not available, wnl: Within normal limits, TPE: Therapeutic plasma exchange, cy: Cycles, PNA: Pneumonia, C: Cervical, IVIG: Intravenous immunoglobulin, kg: Kilogram, MMF: Mycophenolate mofetil, BID: Twice a day, CN VI: Cranial nerve six, b.i.w: Two times a week, wks: Weeks, q. week: Once a week, RTX: Rituximab, J-tube: Jejunostomy feeding tube

Reference	Age	Sex	Symptoms and Physical Exam	Imaging	Stroke Location	Initial Diagnosis	Follow-Up Presentation	Imaging With Location	Laboratory Studies	Diagnosis	Treatment	Notes
Zhou et al., 2015 [7]	56	F	V, Hiccups, dysphagia, hoarseness	MRI brain	Dorsolateral medullary	Ischemic Stroke	L hemiparesis	1) MRI brain: Hypothalamic, periaqueductal, medulla, and cervical medullary. 2) MRI T-spine: wnl	OB+. AQP4-Ab positive (1:10) in blood and CSF	NMOSD	mPRED (1 g/day) x 5d; Oral Steroid taper; AZA	Gradually recovered 3 days after treatment and could walk by herself 20 days later
Asai et al., 2012 [8]	52	F	R sided headache, L hemiparesis and hypesthesia	1) CT brain 2) MRI brain 3) MRA neck 4) Cerebral angiogram	1) R. thalamic hemorrhage 2) Basal ganglia 3) Bilateral distal occlusion of ICA 4) Occlusion of b.l. ACA and MCA	Hemorrhagic stroke; Moya Moya	Hypesthesia of lower (below T6) trunk and limbs, paraplegia, and urinary incontinence. L UE hemiparesis and lower limb paraplegia, +Babinski	MRI spine: T4-T6	OB-. ANA+ (1:80, speckled type), anti–SS-A+, anti–SS-B+, and AQP4-Ab+	NMO	mPRED (1 g/day) x 3d	R sided blindness and partial L sided blindness ~20 years before admission. Paraplegia, sensory disturbance in b.l LE 18 and 15 years before admission that resolved. LUE weakness 5 years before admission. Post mPRED, with improving sensation but continued b.l LE paraplegia.
Cousins et al., 2019 [9]	54	F	N/V, HA, blurry vision, Minimal b.l. palatal rise. No gag reflex. Upbeat nystagmus, paresthesia	1) MRI brain 2) MRA neck	1) Dorsal medulla and upper cervical cord 2) narrowed R VA	Ischemic Stroke with vertebral dissection	N/A	1) MRI brain: posterior cervical-medullary 2) MRI spine: wnl	AQP4-Ab+	NMO	TPE x 5 cy.; mPRED (1 g/day) x 3d; Oral steroid taper (prednisolone)	Treatment paused due to severe PNA. 2 wks post TPE, palatal rise and gag reflex normal
Li et al., 2022 [10]	81	M	L hemiparesis and numbness	N/A	N/A	Ischemic stroke	R hemiparesis and urinary incontinence, +L Babinski	1) MRI brain: wnl 2) MRI spine: extensive T2 hyperintensities from C3-C7 with patchy enhancement	OB-, AQP4-Ab+ (1:32)	NMOSD	mPRED (500 mg/day) x 5d; Oral steroid taper; IVIG (0.4g/kg) x 5d; MMF 750 mg BID upon discharge	Patient died 3 months after discharge due to PNA
Singh et al., 2018 [11]	59	F	L hemiparesis and L shoulder pain 2 days, unsteady gait. L UE dysmetria	MRI brain	Normal	Right hemispheric ischemic stroke	N/A	MRI cervical spine: abnormal enhancement from C3-C4	OB-, AQP4-Ab+ (1:80)	NMOSD	mPRED (1 g/day) x 3d; TPE x 6 cy	Initially thought to have hemorrhagic stroke, then transverse myelitis, then finally NMOSD
Suchdev et al., 2017 [12]	84	F	R numbness and weakness with sensory loss to pinprick and light touch. Diminished LE reflexes	MRI brain	Normal	Ischemic stroke	N/A	MRI spine: C3-T1	OB-. AQP4-Ab+ in both serum and CSF	NMOSD	mPRED (1 g/day) x 5d; TPE x 6 cy; mPRED (1 g/day) x 5d; MMF 500 mg BID, escalated to 1000 mg BID maintenance	Follow up MRI C-spine: reduction in T2 hyper intense lesion at C4–C6 level. 6 months after her initial presentation: increased strength of R UE and decreased numbness
Present Case	58	F	R sided numbness, blurry vision and gait instability	MRI brain	L pons	Ischemic stroke	Constipation, R CN VI palsy, R facial droop, generalized weakness	MRI brain: Brainstem	OB-. AQP4-Ab+ in both serum (1:1000) and CSF (1:32)	NMOSD	IVIG (0.4g/kg) x 5d; mPRED (1 g/day) x 5d, b.i.w for 2 wks, q, week for 4 wks; TPE x 5 cy; RTX x 2 doses	Course first complicated by constipation, later diagnosed with ileus requiring temporary J-tube placement. Required intubation and then tracheostomy.

Notably, brainstem involvement was documented in two out of five cases on the initial brain MRI (no initial brain MRI findings were documented in one case). No abnormalities were detected on the initial brain MRI in two out of five cases. While the stroke neuroimaging findings can initially be absent among patients with stroke, this usually occurs at an interval greater than one month from the acute stroke onset. Thus, the absence of diffusion restriction lesions at the time of presentation with a stroke-like syndrome in these reported cases can also provide clues to alternative etiologies such as brain autoimmunity and/or neuroinflammatory processes. Unfortunately, for some patients, further information regarding which brain hemisphere was affected was not able to be elucidated, reducing further opportunities to correlate infarct location with a presentation [7,9]. 

NMOSD treatment takes the form of two distinct strategies: suppressing the immune system in the acute stage and preventing potential relapses. As demonstrated in the literature and through our own case, steroids, and plasma exchange can often provide an effective therapeutic approach for the management of those affected by NMOSD [20]. Further, some expert opinions argue against delaying plasma exchange and are in favor of combining steroids and plasma exchange to achieve a better outcome [20]. To prevent relapse of the late-onset NMOSD, azathioprine, mycophenolate, or rituximab were used in the reported cases [7,10,12]. Further, a subgroup of AQP4-Ab seropositive patients can benefit from more aggressive immunosuppressive therapies such as those involving terminal complement inhibitors with eculizumab, interleukin-6 inhibition with satralizumab, and next-generation CD19+ B lymphocyte depletion with inebilizumab [20].

## Conclusions

Our case and literature review illustrate that late-onset NMOSD can present as a stroke mimic. Our case underscores the importance of considering the diagnosis of NMOSD in individuals presenting with atypical stroke-like syndromes, particularly those with progressive or recurrent symptoms seemingly related to posterior circulation and/or unexplained comorbid gastrointestinal manifestations. Our review also suggests that this diagnosis should be considered among those with an acute stroke-like presentation lacking the typical acute ischemic changes on the initial brain MRI and vascular imaging. Early recognition and treatment are pivotal to improving functional outcomes, preventing relapses, and limiting mortality.
